# The Association of Olfactory Dysfunction, Frailty, and Mortality Is Mediated by Inflammation: Results from the InCHIANTI Study

**DOI:** 10.1155/2019/3128231

**Published:** 2019-02-20

**Authors:** Alice Laudisio, Luca Navarini, Domenico Paolo Emanuele Margiotta, Davide Onofrio Fontana, Irene Chiarella, Daniele Spitaleri, Stefania Bandinelli, Antonella Gemma, Luigi Ferrucci, Raffaele Antonelli Incalzi

**Affiliations:** ^1^Unit of Geriatrics, Department of Medicine, Università Campus Bio-Medico, Rome, Italy; ^2^Unit of Allergology, Immunology, Rheumatology, Department of Medicine, Università Campus Bio-Medico, Rome, Italy; ^3^Geriatric Rehabilitation Unit, Azienda Sanitaria di Firenze, Florence, Italy; ^4^Department of Homecare Service, Azienda Sanitaria Locale Roma E, Rome, Italy; ^5^Longitudinal Studies Section, Clinical Research Branch, National Institute on Aging, Baltimore, Maryland, USA

## Abstract

**Background:**

Olfactory dysfunction might unveil the association between ageing and frailty, as it is associated with declining cognitive function, depression, reduced physical performance, reduced dietary intake, and mortality; all these conditions are characterized by increased levels of inflammatory parameters. The present study is aimed at evaluating the association between olfactory dysfunction, frailty, and mortality and whether such association might be mediated by inflammation.

**Methods:**

We analysed data of 1035 participants aged 65+ enrolled in the “InCHIANTI” study. Olfactory function was tested by the recognition of the smells of coffee, mint, and air. Olfactory dysfunction was defined as lack of recognition of at least two smells. Considering the items “shrinking,” “exhaustion,” “sedentariness,” “slowness,” and “weakness” included in the Fried definition, frailty was defined as the presence of at least three criteria, prefrailty of one or two, and robustness of none. Serum interleukin-6 (IL-6) was measured in duplicate by high-sensitivity enzyme-linked immunosorbent assays. Logistic regression was adopted to assess the association of frailty with olfactory function, as well as with the increasing number of olfactory deficits. Cox regression was used to test the association between olfactory dysfunction and 9-year survival.

**Results:**

Olfactory dysfunction was associated with frailty, after adjusting (OR 1.94, 95% CI = 1.07-3.51; *P* = .028); analysis of the interaction term indicated that the association varied according to interleukin-6 levels (*P* for interaction = .005). Increasing levels of olfactory dysfunction were associated with increasing probability of being frail. Also, olfactory dysfunction was associated with reduced survival (HR 1.52, 95% CI = 1.16-1.98; *P* = .002); this association varied according to the presence of frailty (*P* for interaction = .017) and prefrailty status (*P* for interaction = .046), as well as increased interleukin-6 levels (*P* for interaction = .011).

**Conclusions:**

Impairment of olfactory function might represent a marker of frailty, prefrailty, and consequently reduced survival in an advanced age. Inflammation might represent the possible link between these conditions.

## 1. Introduction

Due to its prevalence rates exceeding 50% among individuals aged 65-80 years and reaching 80% above the age of 80, olfaction dysfunction is considered a very common problem in older populations [1]. This sensory deficit has important implications for safety, nutrition, quality of life, and social relationships [2]. Olfactory impairment is partially age-related and reflects either central neurodegenerative mechanisms or peripheral cumulative damage of olfactory receptors [1]. In fact, the olfactory system is the only sense which depends upon stem cell turnover, and the olfactory nerve is the only cranial nerve directly exposed to the environment [1].

Frailty is an age-related condition of increased vulnerability, associated with higher risk of several adverse outcomes, including mortality [3]. Among different criteria proposed to define frailty, the frailty phenotype proposed by Fried and colleagues is among the most commonly adopted [4]; also, prefrailty status has been associated with reduced survival, as compared with robustness [3]. Indeed, frailty can be attenuated and even reversed, so that this syndrome has to be considered a dynamic process, mainly for subjects in their intermediate stage [5]. In an Italian cohort of elderly people, although most participants tended to retain their baseline frailty status, more than one-third of the sample experienced a transition (with either improvement or worsening) in their frailty status over a four-year follow-up [6].

It has been documented that sensory perception, including smell perception, is associated with several components of frailty [7]. On the other hand, it has been acknowledged that both frailty and olfactory loss are associated with reduced survival [3, 8]. Furthermore, both olfactory impairment and frailty are characterized by subclinical inflammation, which could partially explain the adverse outcomes associated with these two conditions.

Olfactory impairment, but not hearing or visual impairment, has been associated with decreased survival in older subjects [9]. However, to our knowledge, neither the association of olfactory impairment with prefrailty nor the impact of frailty phenotypes on the association between olfactory dysfunction and mortality has been so far investigated.

The aim of this study was to assess in an older population the association, if any, of olfactory dysfunction with frailty and mortality and whether such an association might be mediated by frailty status.

## 2. Methods

### 2.1. Study Design and Participants

The present study is based upon the data from the “Invecchiare in Chianti” study, a prospective population-based study of older persons in Tuscany, Italy, that is aimed at identifying risk factors for late-life disability [10]. The Italian National Research Council on Aging Ethical Committee ratified the study protocol, and participants provided written consent to participate.

Analyses for this study included all 1035 subjects aged 65+.

### 2.2. Frailty

Frailty was defined according to the Fried criteria [4]: unintended weight loss, self-reported exhaustion, muscle weakness, slowness, and sedentariness. Weight loss was defined as self-reported unintentional weight loss > 4.5 kg within the past year. Exhaustion was defined as a response of “occasionally,” “often,” or “always” to the statement “I felt that everything was an effort.” Muscle weakness was defined as grip strength in the lowest quintile, stratified by sex and BMI quartiles. Grip strength was measured by a handheld dynamometer (Nicholas Muscle Tester, Sammons Preston Inc.). Slowness was defined as the time to walk 4.57 meters or 15 ft (the mean of 2 repetitions) in the slowest quintile, stratified by sex and height. Sedentariness was defined as either complete inactivity or spending <1 h/wk performing low-intensity activities. “Frailty” was defined as the presence of at least three criteria, “prefrailty” of one or two criteria, and “robustness” of none.

This syndrome is thought to emerge from multisystem dysregulation that is common in older adults and characterized by increased vulnerability to stressors and increased risk of disease, disability, and death. Also, frailty is linked to multimorbidity and inflammation.

### 2.3. Mortality

Data on 9-year mortality were collected using the data from the Mortality General Registry maintained by the Tuscany Region, as well as death certificates delivered immediately after death to the registry office of the municipality of residence.

### 2.4. Olfactory Function

Olfactory function was self-reported and explored during the medical visit according to the questions: “Does he/she recognize mint?”, “does he/she recognize coffee?”, and “does he/she recognize air?”. Olfactory dysfunction was defined when at least two smells were not recognized. Increasing levels of olfactory impairment (0 to 3 smell losses) were also considered.

### 2.5. Inflammation

Blood samples were drawn in the morning after a 12-hour overnight fast and resting period. Aliquots of serum were stored at −80°C. Serum interleukin-6 (IL-6) was measured in duplicate by high-sensitivity enzyme-linked immunosorbent assays (ELISAs; kits from BioSource, Camarillo, CA) with a sensitivity of 0.1 pg/mL and an intra-assay coefficient of variations less than 6%.

### 2.6. Covariates

Data on dietary intake were collected by the questionnaire created for the European Prospective Investigation into Cancer and Nutrition (EPIC) study [11]. Adjudicated disease diagnoses were based on self-reported history, clinical documentation, and medication use, as well as standardized criteria derived from the Women's Health and Aging Study protocol [12]. Comorbidity was quantified using the Charlson Comorbidity Index score [13]. All drugs assumed by participants were coded according to the Anatomical Therapeutic Chemical codes [14]. Functional ability was estimated using Katz's activities of daily living [15], depressive symptoms by the original 20-item version of the Center for Epidemiological Studies Depression Scale (CES-D) [16], and cognitive performance by the Mini Mental State Examination [17]. Blood samples were obtained from participants after 12-hour fasting and after resting for at least 15 minutes. Aliquots of serum were stored at –80°C and were not thawed until analysis. Interleukin-6 concentrations were determined by high-sensitivity ELISA using commercial kits (Human Ultrasensitive, BioSource International Inc., Camarillo, CA, USA). Glomerular filtration rate was estimated using the Cockcroft-Gault equation.

### 2.7. Statistical Analyses

Data were recorded using dedicated software. Statistical analyses were performed using Statistical Package for the Social Sciences (SPSS for Mac version 20.0, 2011, SPSS Inc., Chicago, IL); differences were considered significant at the *P* < .050 level.

Data of continuous variables are presented as mean values ± standard deviation or medians and interquartile ranges. Normally distributed variables according to olfactory dysfunction, as well as to mortality, were assessed by the analysis of variance (ANOVA) or the nonparametric Mann–Whitney *U* test if appropriate. The two-tailed Fisher exact test was used for dichotomous variables.

Multivariable logistic regression was used to evaluate the association of the frailty phenotype with age, sex, and all those variables which differed significantly in univariate analysis, including olfactory dysfunction.

The fully adjusted model was also adopted to evaluate the association of increasing levels of olfactory dysfunction with frailty. Also, the analysis of the interaction terms “olfactory dysfunction^∗^interleukin-6” was performed to assess whether the association of frailty with olfactory dysfunction varied according to inflammation.

In addition, to evaluate the whole spectrum of the frailty phenotype, the same summary model was analysed in multinomial logistic regression having robustness, prefrailty, and frailty as the dependent variables.

Also, Cox proportional hazard regression analysis was used to estimate the association of mortality with age, sex, and all those variables which differed significantly in univariate analysis, including olfactory dysfunction. Eventually, in Cox regression, the analysis of the interaction terms “olfactory dysfunction^∗^frailty,” “olfactory dysfunction^∗^prefrailty,” and “olfactory dysfunction^∗^interleukin-6,” was performed to assess whether the association between reduced survival and olfactory dysfunction varied according to the presence of frailty, prefrailty, and inflammatory status.

## 3. Results

The main characteristics of 1035 participants according to olfactory dysfunction are depicted in Table 1. Frailty was diagnosed in 111 (11%) subjects, prefrailty in 420 (41%) participants, and robustness in 504 (48%). The main characteristics of subjects according to frailty are shown in Table 2.

Over the 9-year follow-up, 393 (38%) subjects died. The main characteristics of participants according to survival are depicted in Table 3.

Olfactory dysfunction was reported by 590/1035 (57%) participants; specifically, lack of recognition of one smell was recorded in 190 (18%) subjects, two smells in 243 (23%), and three smells in 347 (33%). In particular, failure to recognize air was found in 638 (62%) subjects, failure to recognize mint was found in 574 (55%), and failure to recognize coffee was found in 505 (49%).

In multivariable logistic regression, olfactory dysfunction was associated with increased probability of being frail (OR 1.94, 95% CI = 1.07-3.51; *P* = .028), after adjusting (Table 4). Analysis of the interaction term indicated that the association of frailty with olfactory dysfunction varied according to interleukin-6 levels (*P* for interaction = .005).

Also, increasing levels of olfactory dysfunction were associated with increasing probability of frailty (*P* for trend = .021).

Both frailty (OR 2.60, 95% CI = 1.39-4.85) and prefrailty (OR 1.59, 95% CI = 1.17-2.16) were associated with olfactory dysfunction in multinomial logistic having robustness as the reference.

**Figure 1:**
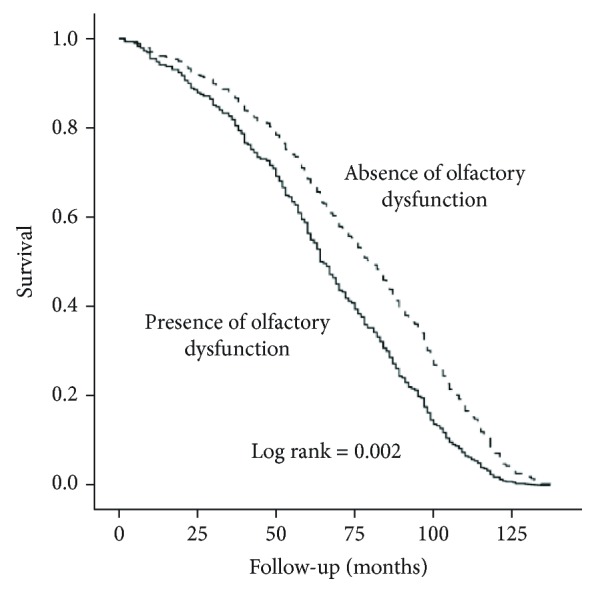
Nine-year survival curves of participants stratified for olfactory dysfunction. The model was simultaneously adjusted for age, sex, education level, glomerular filtration rate, hemoglobin levels, CES-D, Mini Mental State Examination, ADLs, diagnosis of malignancy, peripheral arterial disease, use of ACE inhibitors and benzodiazepines, and frailty.

According to Cox regression analysis, olfactory dysfunction was associated with reduced survival (HR 1.52, 95% CI = 1.16-1.98; *P* = .002), after adjusting (Figure 1); analysis of the interaction term indicated that this association varied according to the presence of frailty (*P* = .017), prefrailty (*P* = .046), and increased interleukin-6 levels (*P* for interaction = .011).

## 4. Discussion

Results of the present study indicate that in older subjects, olfactory dysfunction is associated not only with frailty, but even with prefrailty. This association seems to be mediated by subclinical inflammation.

This association was independent of several confounders, including comorbid conditions, medication use, and lifestyle habits; this finding indicates that olfactory dysfunction might represent an early marker of increased risk for adverse outcomes. In fact, in this population, olfactory dysfunction represented a risk factor for reduced survival, and both frailty and prefrailty seemed to mediate this association.

Several factors might explain the association of olfactory dysfunction with frailty and mortality.

Olfactory dysfunction is among the earliest findings which predict the development of mild cognitive impairment [18]. Also, olfactory dysfunction heralds several neurodegenerative disorders, including Parkinson's disease, which is a paradigm of frailty [2]; of notice, olfactory loss has been included as a marker of prodromal Parkinson's disease by the Movement Disorders Society [19]. Among patients with Parkinson's disease, the severity of olfactory dysfunction seems to correlate with the severity of ensuing dementia [2].

In experimental as well as human models, olfactory dysfunction has been linked with the expression of the apolipoprotein e4 allele and of tau protein and amyloid-*β* deposits [20, 21]; all these findings, in turn, are associated with several adverse clinical outcomes, including cardiovascular diseases and Alzheimer's disease [20]. Olfactory dysfunction has also been associated with depression, probably due to the damage of the hippocampal pathways [22]. In turn, late-life depression has been associated with increased risk of dementia [23].

Systemic diseases, such as diabetes, iron deficiency, and autoimmune diseases, might cause central and peripheral olfactory dysfunction and disrupt the peripheral olfactory pathways [24, 25]. The olfactory deficit represents a preclinical marker of alpha-synucleinopathies and a risk factor for delirium [2, 26]. Also, impairment in olfactory function has been related to the intake of macro- and micronutrients and directly affects food intake behaviour [27]. Eventually, olfactory dysfunction represents a risk factor for reduced survival [8]. Even better, olfactory dysfunction is the only sense which has been associated with mortality, when compared with hearing or visual impairment [9]. With special regard to the frailty components, olfactory function is associated with mobility, balance, fine motor function, and manual dexterity and independent of cognitive function, with challenging upper- and lower-extremity motor function tasks [28]. Also, olfactory loss represents a risk factor for weight loss, while aerobic exercise might preserve olfactory function in selected populations, such as patients with Parkinson's disease [2, 29].

Furthermore, our finding of a potential role of IL-6 serum levels in the association between olfactory loss and frailty is of interest. Increased IL-6 levels have been found in serum and nasal mucus of hyposmic patients [30]. On the other hand, increased IL-6 serum levels have also been associated with frailty, as well as mortality, in older populations [31, 32]. Thus, inflammation represents a common pathophysiological pathway that links hyposmia, frailty, and mortality in the elderly.

In this study, olfactory dysfunction was self-assessed. Self-assessed tools for evaluating olfactory function might underestimate the dysfunction, as compared with objective evaluation. Nevertheless, this would represent a conservative bias, which further supports our findings. Also, regarding the association of olfactory dysfunction with frailty, due to its cross-sectional design, this study does not allow establishing any cause-effect relationship. Nonetheless, this study enrolled a representative community-dwelling population, with high participation rate and with extensive information on risk factors, comorbid conditions, and objective parameters.

In conclusion, olfactory loss represents a correlate of frailty and even of prefrailty; this association seems to affect the role of olfactory dysfunction as a predictor of mortality in older populations. Thus, olfaction seems worth testing in geriatric practice for both clinical and epidemiological purposes.

## Figures and Tables

**Table 1:** Characteristics of 1035 participants according to olfactory dysfunction.

	Presence of olfactory dysfunction (*n* = 590)	Absence of olfactory dysfunction (*n* = 445)	*P*
*Demographics & lifestyle habits,n(%), mean (SD), or median (IQR)*			
Age (years)	76 (8)	73 (7)	<.001
Sex (female)	315 (53)	262 (59)	.088
Education (years)	5 (3)	6 (3)	.003
Living alone	241 (41)	164 (37)	.199
Smoking (former and current)	253 (43)	170 (38)	.142
*Dietary intake*			
Alcohol (g/day/kg)	0.11 (0–0.29)	0.10 (0–0.31)	.594
Total protein intake (g/day/kg)	1.12 (0.33)	1.13 (0.31)	.657
Total lipid intake (g/day/kg)	0.96 (0.31)	0.98 (0.31)	.310
Available carbohydrate intake (g/day/kg)	3.73 (1.17)	3.72 (1.29)	.913
Fibre (g/day/kg)	0.29 (0.08)	0.29 (0.08)	.551
Energy intake (kcal/day/kg)	28.60 (8.25)	28.71 (8.50)	.829
*Comorbid conditions,n(%) or median (IQR)*			
Diabetes	67 (11)	47 (11)	.764
Heart failure	39 (7)	20 (4)	.176
Chronic pulmonary disease	52 (9)	37 (8)	.823
Parkinson's disease	15 (2)	12 (3)	.999
Stroke	37 (6)	23 (5)	.503
Hip fracture	19 (3)	19 (4)	.406
Peripheral arterial disease	80 (14)	41 (9)	.032
Malignancy	28 (5)	36 (8)	.036
Frailty phenotype	85 (14)	26 (6)	<.001
Charlson Comorbidity Index	1 (0-2)	1 (0-1)	.489
*Medications,n(%), mean (SD), or median (IQR)*			
Neuroleptics	18 (3)	15 (3)	.859
Selective serotonin reuptake inhibitors	13 (2)	3 (1)	.072
ACE inhibitors	92 (16)	49 (11)	.035
Antiplatelets	73 (12)	40 (9)	.088
Anticoagulants	8 (1)	5 (1)	.787
Benzodiazepines	112 (19)	61 (14)	.029
Loop diuretics	53 (9)	32 (7)	.306
Corticosteroids	8 (1)	10 (2)	.339
*Biohumoral, physical, and cognitive parameters,n(%) ormean* ± *SD*			
Glomerular filtration rate (mL/min)	62.6 (19.3)	68.1 (19.2)	<.001
Total serum proteins (g/dL)	7.2 (0.4)	7.1 (0.5)	.308
Interleukin 6 (pg/mL)	1.49 (0.84-2.32)	1.44 (0.88-2.27)	.582
Hemoglobin (g/dL)	13.6 (1.4)	13.8 (1.4)	.112
CES-D	13 (9)	12 (8)	.035
Mini Mental State Examination	24.3 (4.3)	24.7 (5.5)	.160
Katz's activities of daily living	5 (1)	4 (2)	.107
Body mass index (kg/m^2^)	27.3 (4.0)	27.6 (4.2)	.183

**Table 2:** Characteristics of 1035 participants according to the presence of frailty.

	Positive for frailty (*n* = 111)	Negative for frailty (*n* = 924)	*P*
*Demographics & lifestyle habits,n(%), mean (SD), or median (IQR)*			
Age (years)	81 (7)	74 (7)	<.001
Sex (female)	70 (63)	507 (55)	.106
Education (years)	4 (3)	5 (3)	<.001
Living alone	68 (61)	337 (36)	<.001
Smoking (former and current)	38 (34)	385 (42)	.153
*Dietary intake*			
Alcohol (g/day/kg)	0.04 (0–0.25)	0.11 (0–0.31)	.021
Total protein intake (g/day/kg)	1.04 (0.30)	1.12 (0.32)	.009
Total lipid intake (g/day/kg)	0.87 (0.28)	0.97 (0.31)	.002
Available carbohydrates intake (g/day/kg)	3.41 (1.11)	3.76 (1.23)	.008
Fibre (g/day/kg)	0.27 (0.09)	0.30 (0.09)	.006
Energy intake (kcal/day/kg)	26.0 (7.5)	29.0 (8.4)	.001
*Comorbid conditions,n(%) or median (IQR)*			
Diabetes	16 (14)	98 (11)	.259
Heart failure	19 (17)	40 (4)	<.001
Chronic pulmonary disease	18 (16)	71 (8)	.006
Parkinson's disease	8 (7)	19 (2)	.002
Stroke	16 (14)	44 (5)	<.001
Hip fracture	11 (10)	27 (3)	.001
Peripheral arterial disease	23 (21)	98 (11)	.004
Malignancy	8 (7)	56 (6)	.675
Olfactory dysfunction	85 (77)	505 (55)	<.001
Charlson Comorbidity Index	1 (1-2)	0 (0-1)	<.001
*Medications,n(%), mean (SD), or median (IQR)*			
Neuroleptics	10 (9)	23 (5)	.001
Selective serotonin reuptake inhibitors	7 (6)	9 (1)	.001
ACE inhibitors	24 (22)	117 (13)	.013
Antiplatelets	18 (16)	95 (10)	.075
Anticoagulants	2 (2)	11 (1)	.641
Benzodiazepines	32 (29)	141 (15)	.001
Loop diuretics	21 (19)	64 (7)	<.001
Corticosteroids	4 (4)	14 (1)	.118
*Biohumoral, physical, and cognitive parameters,n(%) ormean* ± *SD*			
Glomerular filtration rate (mL/min)	54.8 (20.5)	66.0 (19.0)	<.001
Total serum proteins (g/dL)	7.2 (0.6)	7.1 (0.4)	.838
Interleukin 6 (pg/mL)	2.21 (1.35 – 4.09)	1.40 (0.83–2.07)	<.001
Hemoglobin (g/dL)	13.1 (1.6)	13.8 (1.3)	<.001
CES-D	20 (9)	12 (8)	<.001
Mini Mental State Examination	21 (6)	25 (4)	<.001
Katz's activities of daily living	6 (0-1)	6 (0–0)	<.001
Body mass index (kg/m^2^)	27.9 (5.1)	27.4 (4.0)	.289

**Table 3:** Characteristics of 1035 participants according to survival status.

	Dead (*n* = 393)	Alive (*n* = 642)	*P*
*Demographics & lifestyle habits,n(%), mean (SD), or median (IQR)*			
Age (years)	80 (7)	72 (5)	<.001
Sex (female)	200 (51)	377 (59)	.014
Education (years)	5 (3)	6 (3)	<.001
Living alone	206 (52)	199 (31)	<.001
Smoking (former and current)	172 (44)	251 (39)	.152
*Dietary intake*			
Alcohol (g/day/kg)	0.10 (0–0.26)	0.10 (0–0.32)	.021
Total protein intake (g/day/kg)	1.14 (0.32)	1.11 (0.32)	.262
Total lipid intake (g/day/kg)	0.97 (0.30)	0.96 (0.32)	.582
Available carbohydrate intake (g/day/kg)	3.82 (1.26)	3.67 (1.20)	.066
Fibre (g/day/kg)	0.29 (0.09)	0.29 (0.08)	.654
Energy intake (kcal/day/kg)	29.08 (8.33)	28.40 (8.37)	.227
*Comorbid conditions,n(%) or median (IQR)*			
Diabetes	52 (13)	62 (10)	.082
Heart failure	46 (12)	13 (2)	<.001
Chronic pulmonary disease	63 (16)	26 (4)	<.001
Parkinson's disease	21 (5)	6 (1)	<.001
Stroke	44 (11)	16 (2)	<.001
Hip fracture	24 (6)	14 (2)	.002
Peripheral arterial disease	86 (22)	35 (5)	<.001
Malignancy	28 (7)	36 (6)	.353
Olfactory dysfunction	249 (63)	341 (53)	.001
Frailty	90 (23)	21 (3)	<.001
Charlson Comorbidity index	1 (0-2)	0 (0-1)	<.001
*Medications,n(%), mean (SD), or median (IQR)*			
Neuroleptics	17 (4)	16 (2)	.143
Selective serotonin reuptake inhibitors	11 (3)	5 (1)	.017
ACE inhibitors	72 (18)	69 (11)	.001
Antiplatelets	65 (16)	48 (7)	<.001
Anticoagulants	11 (3)	2 (1)	.001
Benzodiazepines	81 (21)	92 (14)	.010
Loop diuretics	55 (14)	30 (5)	<.001
Corticosteroids	11 (3)	7 (1)	.050
*Biohumoral, physical, and cognitive parameters,n(%) ormean* ± *SD*			
Glomerular filtration rate (mL/min)	56.4 (19.5)	69.5 (17.8)	<.001
Total serum proteins (g/dL)	7.1 (0.5)	7.1 (0.4)	.680
Interleukin 6 (pg/mL)	1.89 (1.14–3.31)	1.22 (0.78–1.84)	<.001
Hemoglobin (g/dL)	13.4 (1.6)	13.9 (1.2)	<.001
CES-D	14 (9)	12 (9)	<.001
Mini Mental State Examination	22 (6)	26 (3)	<.001
Katz's activities of daily living	5 (1)	6 (0)	<.001
Body mass index (kg/m^2^)	27.0 (4.3)	27.7 (4.0)	.016

**Table 4:** Association (odds ratios (OR) and 95% confidence intervals (CI)) of frailty with the variables of interest, including olfactory dysfunction, according to the logistic regression model. All the covariates were entered simultaneously into the regression model.

	OR	95% CI	*P*
Age (each year)	1.15	1.09-1.21	<.001
Sex (female)	.92	.52-1.64	.778
Education (years)	.95	.86-1.05	.306
Malignancy	1.72	.65-4.57	.279
Peripheral arterial disease	2.46	1.29-4.69	.006
ACE inhibitors	1.65	.87-3.12	.122
Benzodiazepines	1.15	.63-2.10	.653
Glomerular filtration rate (mL/min)	1.02	1.01-1.04	.018
CES-D	1.11	1.07-1.14	.000
Olfactory dysfunction	1.94	1.07-3.51	.028

## Data Availability

The data used to support the findings of this study are available from the corresponding author upon request.
